# Clinical presentation and treatment response in patients with polymyalgia rheumatica and giant cell arteritis during a 40-week follow-up

**DOI:** 10.1093/rap/rkab091

**Published:** 2021-11-24

**Authors:** Amir Emamifar, Søren Hess, Torkell Ellingsen, Oke Gerke, Ziba Ahangarani Farahani, Per Syrak Hansen, Inger Marie Jensen Hansen, Peter Thye-Rønn

**Affiliations:** 1 Department of Clinical Research, Faculty of Health Sciences, University of Southern Denmark, Odense; 2 Diagnostic Center; 3 Department of Rheumatology, Svendborg Hospital, OUH, Svendborg; 4 OPEN, Odense Patient data Explorative Network, Odense University Hospital, Odense; 5 Department of Radiology and Nuclear Medicine, Hospital of Southwest Jutland, Esbjerg; 6 Institute of Regional Health Research, Faculty of Health Sciences, University of Southern Denmark; 7 Rheumatology Research Unit, Odense University Hospital and University of Southern Denmark; 8 Research Unit of Clinical Physiology and Nuclear Medicine, Department of Clinical Research, University of Southern Denmark; 9 Department of Nuclear Medicine, Odense University Hospital, Odense, Denmark

**Keywords:** Polymyalgia rheumatica, giant cell arteritis, treatment response, ^18^F-fluorodeoxyglucose PET/CT, temporal artery biopsy

## Abstract

**Objectives:**

The aim was to study the clinical features of PMR/GCA and clinical predictors of treatment response during a 40-week follow-up period.

**Methods:**

Clinical data on 77 patients with newly diagnosed PMR/GCA who were treated with oral glucocorticoids were gathered at baseline and during a 40-week follow-up period. A unilateral temporal artery biopsy (TAB) and ^18^F-fluorodeoxyglucose (^18^F-FDG) PET/CT were undertaken at diagnosis. In total, each patient was seen on five occasions (i.e. baseline and weeks 4, 16, 28 and 40). Treatment response was assessed by considering clinical evaluations and results of inflammatory markers.

**Results:**

Of 77 patients [49 (63.6%) female; mean age 71.8 (8.0) years], 64 (83.1%) patients had pure PMR, 10 (13.0%) concomitant PMR and GCA, and 3 (3.9%) pure GCA. The patients reported that clinical symptoms, apart from scalp pain and duration of morning stiffness, improved significantly at week 4 and remained lower at week 40 compared with the relative frequencies at baseline. Besides, all components of physical examination showed significant improvement and remained lower at week 40 compared with the baseline. A complete response was seen in 68.7, 62.9, 44.1 and 33.3% of patients at weeks 4, 16, 28 and 40, respectively. Several clinical features, including female biological sex, younger age, fewer relapses and a lower level of baseline ESR, were significantly associated with a better treatment response. Treatment response during the follow-up period was independent of TAB results and fluorodeoxyglucose uptakes on ^18^F-FDG PET/CT at diagnosis.

**Conclusion:**

Obtaining valid disease-specific outcome measures for evaluating treatment efficacy in PMR and GCA that can be applied universally is clearly an unmet clinical need.

**Trial registration:**

ClinicalTrials.gov, https://clinicaltrials.gov, NCT02985424.

Key messages

- Contrary to the cranial symptoms, constitutional and shoulder/hip girdle symptoms deteriorate by tapering prednisolone.
- Treatment response in PMR/GCA is independent of temporal artery biopsy results and fluorodeoxyglucose uptakes on ^18^F-fluorodeoxyglucose PET/CT.
- Obtaining valid disease-specific outcome measures for evaluating treatment efficacy in PMR/GCA is essential.

## Introduction

PMR is the most common inflammatory rheumatic disease of the elderly and typically presents with pain and stiffness in the neck, shoulder girdle and hip girdle [1]. GCA, the most remarkable association with PMR, is a medium-large vessel vasculitis with ischaemic manifestations of the involved vessels [2]. Although there is no consensus among researchers, several authors believe that PMR and GCA are part of the same disease entity, whereby PMR is at one end of disease spectrum and GCA at the other. Accordingly, imaging findings support this model [3]. Constitutional symptoms such as fever, weight loss and tiredness, although unspecific, are commonly seen in PMR and GCA and could be associated with the activity of the inflammatory disease [2, 4]. Glucocorticoids remain the mainstay of treatment, and resolution of symptoms following treatment initiation has been considered as a diagnostic hallmark [5]. Nevertheless, there is little hard evidence on what constitutes an appropriate response in patients, how treatment response contributes to long-term outcomes during and after glucocorticoid therapy, and which outcomes should be monitored. Besides, PMR and GCA are subject to wide variations in clinical practice that make it challenging to identify the factors associated with favourable outcome, particularly a sustained remission.

To date, longitudinal data on clinical features of PMR and/or GCA (PMR/GCA) and outcome predictors are limited. Whether there is a pattern to determine long-term outcomes is not well understood, and clarifying the predictors of disease activity and disease outcomes is an unmet need. Recently, we published the results of a cohort study on 77 patients newly diagnosed PMR and GCA, with a focus on the diagnostic utility of ^18^F-fluorodeoxyglucose (^18^F-FDG) PET/CT [6]. To secure a confident diagnosis, patients were followed for 40 weeks, while their clinical symptoms and signs were closely monitored and gathered. In line with these considerations, the aim of the present study was to investigate the clinical features of the PMR/GCA patients during the 40-week study period. Furthermore, the clinical predictors of treatment response at weeks 4, 16, 28 and 40 were examined.

## Method

### Study design and setting

This is a prospective cohort study. The study was conducted at the Diagnostic Center in collaboration with the section of Rheumatology, Svendborg Hospital, between February 2018 and December 2019. Ethical approval was sought from the Regional Ethics committee of the Region of Southern Denmark (identification number: S-20160098) and the Danish Data Protection Agency (J.nr 16/40522). The study was also registered at ClinicalTrials.gov (identifier: NCT02985424).

### Participants

Seventy-seven consecutive patients with newly diagnosed PMR, GCA, or concomitant PMR and GCA were followed for 40 weeks after inclusion in the study. Written informed consent was initially obtained from all patients. Inclusion and exclusion criteria have been published previously in the protocol [6, 7]. Briefly, five components of the following criteria were satisfied to suspect PMR: age ≥50 years, bilateral shoulder or hip pain, morning stiffness lasting >45 min, elevated ESR, elevated CRP and disease duration >2 weeks. To suspect cranial GCA, the following criteria were considered: age ≥50 years and elevated ESR/CRP, together with at least two symptoms related to vasculitis [scalp tenderness, vision disturbances, headache (new or changed), jaw claudication and tenderness of the temporal arteries]. One cranial symptom was enough to suspect cranial GCA in those with concomitant PMR. Patients with clinical suspicion of large vessel GCA were also eligible for inclusion. Patients were excluded from the study if they met one of the following criteria: (1) infections, malignancy or any other conditions in which prednisolone was permanently unsuitable; (2) contraindication to ^18^F-FDG PET/CT (blood glucose ≥145 mg/dl after 6 h fasting); (3) initiation of glucocorticoid treatment >3 days before ^18^F-FDG PET/CT; (4) inability to provide informed consent; and (5) dementia or inability to communicate in Danish. In total, each included patient was seen on five occasions [i.e. at baseline (visit 1), week 4 (visit 2), week 16 (visit 3), week 28 (visit 4) and week 40 (visit 5)]. Oral doses of 20–30 mg/day and ≤75 mg/day oral prednisolone were initially used to treat PMR and GCA, respectively [8]. Telephone follow-up was conducted by a rheumatology specialist nurse between each physician consultation, and patients were asked to contact the outpatient clinic in the event of a deterioration of the symptoms.

### Data collection

Patients’ clinical symptoms, including constitutional symptoms (weight loss, tiredness and fever), shoulder girdle (pain in neck, shoulder or upper arm), hip girdle (pain in buttock and thigh), morning stiffness (in minutes) and cranial region (scalp pain, new/changed headache, jaw claudication, visual disturbances or temporal area pain), and physical examination (neck tenderness to palpation, shoulder tenderness to palpation, upper arm tenderness to palpation, active shoulder abduction, buttock tenderness to palpation, thigh tenderness to palpation, temporal artery tenderness to palpation and scalp tenderness to palpation) at baseline and follow-up visits were collected and managed by means of Research Electronic Data Capture (REDcap), which is a secure, Web-based software platform [9]. Patients were urgently referred to ophthalmologists if there was any suspicion of visual disturbances caused by GCA.

Treatment response at follow-up visits was defined as complete response if all three of the following were met: (1) ≥70% improvement in PMR/GCA global visual analogue scale (VAS; scores range from 0 to 100 mm); (2) normal CRP (normal value <6.0 mg/l) and ESR (normal value 2–20 mm); and (3) ≥70% reduction in duration of morning stiffness if there was any; partial response if two of the three were met; and non-response if none or one of the three was met [10]. For GCA, in addition to the mentioned criteria, no signs of the recurrence of GCA symptoms and a need for an increase in the prednisolone dose were also satisfied, as assessed individually by the treating physician [11]. PMR/GCA global VAS scores were derived based on the patients’ responses to the following question: on a scale from 0 (no effect) to 100 (maximum effect), how would you rate how your PMR/GCA affects you/your health in the last 2 days? Relapse was defined as recurrence of the symptoms or signs of the disease (pain and stiffness in shoulder and/or hip girdles, morning stiffness, shoulder and/or hip tenderness to palpitation, limitation of upper limb elevation in the case of PMR, and jaw claudication, visual disturbances, scalp pain or tenderness to palpitation, temporal area pain or tenderness to palpitation, limb claudication in the case of GCA) together with the elevation of acute phase reactants.

Furthermore, a unilateral temporal artery biopsy (TAB) and ^18^F-FDG PET/CT in all patients were also undertaken at baseline. TAB was considered positive if signs of active arteritis or healed arteritis were detected on histopathological examination. ^18^F-FDG PET/CT scans were described visually based on a four-point visual grading scale (VGS) considering two pathological cut-off values of VGS ≥ 3 and VGS ≥ 2 [6]. Results of ^18^F-FDG PET/CT were categorized into the following: neither PMR nor GCA activity; PMR activity; GCA activity; or PMR and GCA activity. Total PMR and GCA scores were derived from the sum of VGS in each articular/periarticular site and arterial segment, respectively.

### Statistical analysis

Data are presented as frequencies (percentages), the mean (S.d.) or the median [interquartile range (IQR)] depending on the type of data and distribution. A comparison of paired and unpaired categorical variables was performed using McNemar’s test and Fisher’s exact test, respectively. Continuous variables were compared by Student’s *t*-test or Wilcoxon rank-sum test (Mann–Whitney *U*-test), if unpaired, and Wilcoxon signed-rank test, if paired. The Kruskal–Wallis test or analysis of variance was used when more than two groups were compared. Frequencies of clinical symptoms (i.e. constitutional symptoms, shoulder girdle and hip girdle symptoms and cranial symptoms) are shown using a stacked bar graph. Results of the patients’ global VAS, ESR, CRP and duration of morning stiffness, in addition to prednisolone treatment at baseline, weeks 4, 16, 28 and 40, are shown graphically by means of box plots displaying the median, 25th and 75th percentiles, minimum and maximum values within the fences 25th percentile minus 1.5 × IQR and 75th percentile plus 1.5 × IQR, and outliers. Treatment response (i.e. complete, partial or non-response) with relative frequencies for each group at weeks 4, 16, 28 and 40 are illustrated using a bar graph. Data for the subgroup analysis on patients with pure PMR are presented in Supplementary Materials, available at *Rheumatology Advances in Practice* online. A *P*-value was considered significant if *P* < 0.05. No method of imputation was used for missing data. Statistical analysis was performed using STATA v.16.0 (StataCorp, College Station, TX, USA).

## Results

**Fig. 1:**
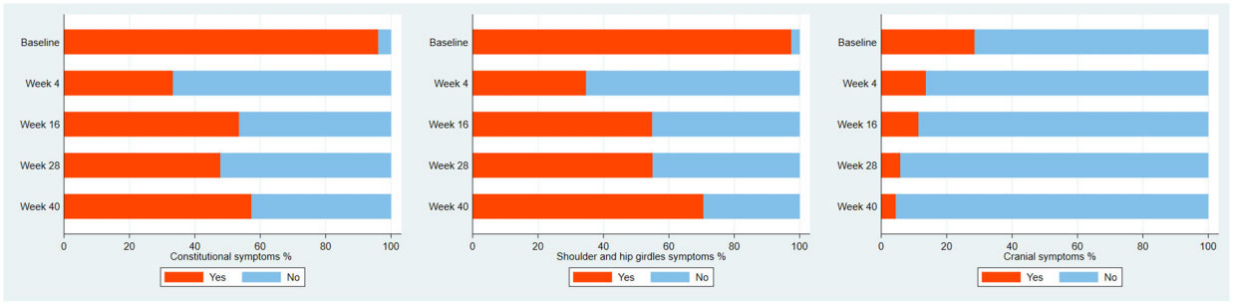
Variation of clinical symptoms during the study period

Of 77 included patients, 64 (83.1%) patients were diagnosed with pure PMR, 10 (13.0%) patients with concomitant PMR and GCA, and 3 (3.9%) with pure GCA. Forty-nine (63.6%) patients were female, and mean age was 71.8 (8.0) years. Four (5.2%) patients developed cancer during the study period [i.e. breast cancer (*n* = 2), adenocarcinoma of the colon (*n* = 1) and adenocarcinoma of colon together with skin cancer (*n* = 1)] and four (5.2%) were positive for monoclonal gammopathy of undetermined significance [12]. Baseline demographic data have been published previously in detail [6]. Total number of relapses during the study and cumulative prednisolone dose (in milligrams) were as follows: mean (S.d.) 0.6 (0.9), median (IQR) 0 (0–1), minimum 0 and maximum 5; and mean (S.d.) 2995.1 (1269.7), median (IQR) 2438.7 (2193.7–3420), minimum 1226.25 and maximum 7511.25, respectively. The daily prednisolone dose at baseline and during follow-up together with the cumulative prednisolone dose during follow-up are depicted in Supplementary Figs S1 and S2, available at *Rheumatology Advances in Practice* online. The numbers of patients who completed visit 2 (week 4) were 73, visit 3 (week 16) 71, visit 4 (week 28) 69 and visit 5 (week 40) 69. Altogether, 69 patients completed all five visits. Reasons for withdrawal were as follows: not interested (*n* = 5), lost to follow-up (*n* = 1) and prednisolone side-effects (*n* = 1). Besides, one patient died because of a ruptured abdominal aortic aneurism. A summary of clinical symptoms together with physical examinations at baseline and follow-up visits at weeks 4, 16, 28 and 40 are shown in Tables 1 and 2 and in Supplementary Tables S1 and S2, available at *Rheumatology Advances in Practice* online. Although 14 (18.2%) patients complained about visual disturbances, no visual changes caused by GCA were confirmed on physical examinations. Patients’ clinical symptoms [i.e. constitutional, shoulder and hip girdles, morning stiffness (frequency) and cranial], apart from scalp pain and duration of morning stiffness, improved significantly after initiation of treatment, at week 4, and remained lower at week 40 compared with the relative frequencies at baseline (Table 1). Besides, all components of physical examination showed significant improvement and remained lower at week 40 compared with the baseline (Table 2). However, the variation of constitutional and shoulder girdle and hip girdle symptoms, contrary to the cranial symptoms, showed gradual deterioration in the symptoms along with the reduction of prednisolone dose, when associated symptoms were considered in combination (Fig. 1; Supplementary Fig. S3, available at *Rheumatology Advances in Practice* online). At least one constitutional symptom was seen in 57.3% of the patients; 70.6% of patients presented with one in either the shoulder or hip girdle, and 4.5% with one cranial symptom at week 40.

**Table 1:** Summary of patient-reported symptoms at baseline and weeks 4, 16, 28 and 40

Symptoms	Baseline (*n* = 77)	Week 4 (*n* = 73)	Week 16 (*n* = 71)	Week 28 (*n* = 69)	Week 40 (*n* = 69)
Constitutional, *n* (%)					
Weight loss	30 (39.0)	8 (11.0), **<0.001^a^**	4 (5.6), **<0.001^a^**	1 (1.4), **<0.001^a^**	10 (14.7), **0.001^a^**
Tiredness	73 (94.8)	22 (30.6), **<0.001^a^**	34 (47.9), **<0.001^a^**	31 (44.9), **<0.001^a^**	36 (52.9), **<0.001^a^**
Fever	17 (22.1)	0 (0), **<0.001^a^**	3 (4.2), **<0.001^a^**	2 (2.9), **<0.001^a^**	0 (0), **<0.001^a^**
Shoulder girdle, *n* (%)					
Pain in neck	51 (67.1)	8 (11.0), **<0.001^a^**	14 (19.7), **<0.001^a^**	8 (11.6), **<0.001^a^**	14 (20.6), **<0.001^a^**
Pain in shoulder	68 (88.3)	7 (9.6), **<0.001^a^**	16 (22.5), **<0.001^a^**	13 (18.8), **<0.001^a^**	25 (36.8), **<0.001^a^**
Pain in upper arm	67 (87.0)	7 (9.7), **<0.001^a^**	15 (21.1), **<0.001^a^**	15 (21.7), **<0.001^a^**	17 (25.0), **<0.001^a^**
Hip girdle, *n* (%)					
Pain in buttock	63 (81.8)	12 (16.4), **<0.001^a^**	15 (21.1), **<0.001^a^**	15 (21.1), **<0.001^a^**	15 (21.1), **<0.001^a^**
Pain in thigh	63 (82.9)	7 (9.6), **<0.001^a^**	15 (21.1), **<0.001^a^**	16 (23.2), **<0.001^a^**	17 (25.0), **<0.001^a^**
Cranial, *n* (%)					
Scalp pain	7 (9.1)	3 (4.1), 0.10^a^	2 (2.9), 0.10^a^	0 (0), **0.014^a^**	1 (1.5), 0.06^a^
New/changed headache	15 (19.5)	2 (2.7), **<0.001^a^**	4 (5.6), **0.020^a^**	2 (2.9), **0.003^a^**	0 (0), **<0.001^a^**
Jaw claudication	10 (13.0)	1 (1.4), **0.003^a^**	1 (1.4), **0.005^a^**	2 (2.9), **0.020^a^**	1 (1.5), **0.005^a^**
Visual disturbances	14 (18.2)	5 (6.8), **0.011^a^**	3 (4.2), **0.007^a^**	1 (1.4), **<0.001^a^**	2 (2.9), **0.004^a^**
Temporal area pain	9 (11.7)	1 (1.4), **0.011^a^**	2 (2.8), 0.06^a^	0 (0), **0.008^a^**	1 (1.5), **0.014^a^**
Morning stiffness, *n* (%)	63 (82.9)	6 (8.2), **<0.001^a^**	16 (22.5), **<0.001^a^**	27 (39.7), **<0.001^a^**	32 (47.1), **<0.001^a^**
Morning stiffness, min	60 (30–120)	60 (30–120), 0.32^b^	45 (10–60), 0.21^b^	30 (30–60), 0.36^b^	60 (30–120), 0.91^b^

The *P*-values relate to hypothesis testing on the difference between baseline and follow-up data. Bold values indicate statistically significant differences. ^a^McNemar’s test. ^b^Wilcoxon signed-rank test.

**Table 2:** Summary of physical examination at baseline and weeks 4, 16, 28 and 40

Physical examination, *n* (%)	Baseline	Week 4	Week 16	Week 28	Week 40
Neck tenderness to palpation	15 (22.4)	2 (2.7), **<0.001**	2 (2.9), **<0.001**	3 (4.3), **<0.001**	2 (3.0), **0.001**
Shoulder tenderness to palpation	18 (26.9)	3 (4.1), **<0.001**	5 (7.2), **0.003**	4 (5.9), **<0.001**	2 (3.0), **<0.001**
Upper arm tenderness to palpation	28 (41.8)	2 (2.7), **<0.001**	3 (4.3), **<0.001**	7 (10.1), **<0.001**	3 (4.5), **<0.001**
Active shoulder abduction, 0–180°					
Not at all	19 (27.9)	0 (0)	1 (1.4)	2 (2.9)	2 (3.0)
Yes, hardly	41 (60.3)	4 (5.7)	1 (1.4)	3 (4.3)	7 (10.4)
Yes, effortless	8 (11.8)	66 (94.3), **<0.001**	68 (97.1), **<0.001**	64 (92.7), **<0.001**	58 (86.6), **<0.001**
Buttock tenderness to palpation	23 (34.3)	6 (8.2), **<0.001**	6 (8.7), **<0.001**	2 (2.9), **<0.001**	0 (0), **<0.001**
Thigh tenderness to palpation	25 (37.3)	4 (5.5), **<0.001**	6 (8.7), **<0.001**	2 (2.9), **<0.001**	3 (4.5), **<0.001**
Temporal artery tenderness to palpation	5 (8.1)	2 (3.1), 0.18	5 (7.5), **0.045**	2 (3.1), 0.08	0 (0), **0.045**
Scalp tenderness to palpation	4 (6.0)	1 (1.4), 0.10	0 (0), 0.99	1 (1.4), 0.10	0 (0), **0.025**

The *P*-values were calculated by McNemar’s test and relate to hypothesis testing on the difference between baseline and follow-up data.Bold values indicate statistically significant differences.

With reference to the previously published results, TAB was positive in 7 (10%) patients [i.e. active arteritis, *n* = 4 (5.7%), and healed arteritis, *n* = 3 (4.3%)]. Regarding ^18^F-FDG PET/CT VGS, when a pathological cut-off value of ≥3 was considered pathological, 55 (71.4%) patients had signs of PMR activity, 2 (2.6%) GCA activity, 1 (1.3%) PMR and GCA activity, and 19 (24.7%) did not display any PMR or GCA activity. When a cut-off value of ≥2 was considered pathological, 58 (75.3%) patients showed PMR activity, 3 (3.9%) GCA activity, 9 (11.7%) PMR and GCA activity, and 7 (9.1%) did not display any PMR or GCA activity. Out of 64 patients with the clinical diagnosis of pure PMR, 1 (1.6%) (pathological uptake cut-off of ≥3) and 6 (9.4%) (pathological uptake cut-off of ≥2) patients showed signs of vasculitis on ^18^F-FDG PET/CT, depending on the cut-off values used to define the pathological uptakes. The total PMR and GCA scores were as follows: mean (S.d.) 12.5 (5.9), median (IQR) 14 (10–17), minimum 0 and maximum 21; and mean (S.d.) 0.8 (2.0), median (IQR) 0 (0–0), minimum 0 and maximum 12, respectively [6]. Comparison of clinical data, laboratory measures, TAB and imaging results in patients with and without constitutional, shoulder and hip girdle and cranial symptoms at week 40 are summarized in Table 3 and Supplementary Table S3, available at *Rheumatology Advances in Practice* online, to the extent that data were available.

**Table 3:** Comparison of data in patients with and without constitutional, shoulder and hip girdle and cranial symptoms at week 40

	Week 40								
Variables	Any constitutional symptoms			Any shoulder and hip girdles symptoms			Any cranial symptoms		
	No	Yes	*P*-value	No	Yes	*P*-value	No	Yes	*P*-value
Age, mean (S.d.), years	71.1 (7.6)	71 (7.3)	0.94^a^	70.2 (8.4)	71.4 (7.0)	0.54^a^	71.2 (7.3)	65.7 (9.3)	0.21^a^
Female gender, *n* (%)	18 (26.5)	25 (36.8)	0.99^b^	14 (20.6)	29 (42.6)	0.58^b^	41 (61.2)	2 (3.0)	0.99^b^
Baseline BMI, kg/m^b^	24.1 (21.4–26.9)	25.8 (23–28.3)	0.16^c^	24.2 (21.7–27.4)	25.5 (22.2–28.1)	0.41^c^	25.3 (22.0–27.9)	26.9 (25.3–29.8)	0.25^c^
Charlson co-morbidity index	3 (2–4)	3 (3–4)	0.18^c^	4 (3–5)	3 (2–4)	0.13^c^	3 (2–4)	3 (1–4)	0.51^c^
Clinical diagnosis			0.70^b^			**0.016^b^**			0.58^b^
Pure PMR	24 (35.3)	34 (50.0)		**14 (20.6)**	**44 (64.7)**		55 (82.1)	2 (3.0)	
Pure GCA	0 0	1 (1.5)		**0 0**	**1 (1.5)**		1 (1.5)	0 0	
Concomitant PMR and GCA	5 (7.3)	4 (5.9)		**6 (8.8)**	**3 (4.4)**		8 (11.9)	1 (1.5)	
Smoker (including former smoker), *n* (%)	16 (23.5)	29 (42.6)	0.12^b^	13 (19.1)	32 (47.1)	0.99^b^	42 (62.7)	2 (3.0)	0.99^b^
Alcohol > 6 units/week	6 (8.8)	14 (20.6)	0.19^b^	4 (5.9)	16 (23.5)	0.38^b^	19 (28.4)	1 (1.5)	0.99^b^
Baseline ESR, mm [normal range: 2–20]	56 (37–77)	53 (40–73)	0.95^c^	60 (27–87)	51 (40–73)	0.75^c^	**53 (38–75)**	**88 (83–98)**	**0.019^c^**
Baseline CRP, mg/l [normal range: <6.0]	44 (25–71)	33 (15–60)	0.11^c^	**56 (34–91.5)**	**31 (16.5–59)**	**0.013^c^**	**37 (17.5–60)**	**83 (76–173)**	**0.018^c^**
Baseline fibrinogen, µmol/l [normal range: 5.2–12.6]	15.1 (13.1–17.6)	14.9 (12.8–17.2)	0.59^c^	15.3 (12.9–17.9)	14.9 (13–17.2)	0.72^c^	15 (13–17.2)	20.6 (2.2–21.7)	0.53^c^
Temporal artery biopsy, *n* (%)			0.13^b^			0.31^b^			0.99^b^
Positive	0 0	4 (6.4)		0 0	4 (6.9)		3 (4.9)	0 0	
Negative	26 (41.9)	32 (51.6)		18 (29.0)	40 (64.5)		55 (90.2)	3 (4.9)	
^18^F-Fluorodeoxyglucose PET/CT cut-off ≥3, *n* (%)			**0**			0.37^b^			0.99^b^
Neither PMR nor GCA activity	**3 (4.4)**	**14 (20.6)**	**0**	6 (8.8)	11 (16.2)		16 (23.9)	1 (1.5)	
PMR activity	**26 (38.2)**	**23 (33.8)**	**1**	13 (19.1)	36 (52.9)		46 (68.7)	2 (3.0)	
GCA activity	**0 0**	**1 (1.5)**	**1**	1 (1.5)	0 0		1 (1.5)	0 0	
PMR and GCA activity	**0 0**	**1 (1.5)**	**^b^**	0 0	1 (1.5)		1 (1.5)	0 0	
^18^F-Fluorodeoxyglucose PET/CT cut-off ≥2, *n* (%)			0.72^b^			0.10^b^			0.19^b^
Neither PMR nor GCA activity	2 (2.9)	5 (7.3)		3 (4.4)	4 (5.9)		7 (10.4)	0 0	
PMR activity	24 (35.3)	28 (41.2)		14 (20.6)	38 (55.9)		49 (73.1)	2 (3.0)	
GCA activity	1 (1.5)	1 (1.5)		2 (2.9)	0 0		1 (1.5)	1 (1.5)	
PMR and GCA activity	2 (2.9)	5 (7.3)		1 (1.5)	6 (8.8)		7 (10.4)	0 0	
PMR score	15 (12–17)	13 (7–17)	0.26^c^	12.5 (8–16.5)	14 (10–17)	0.52^c^	14 (10–17)	12 (6–15)	0.39^c^
GCA score	0 (0–0)	0 (0–0)	0.50^c^	0 (0–0)	0 (0–0)	0.63^c^	0 (0–0)	0 (0–6)	0.38^c^
Cumulative prednisolone dose, mg	2305 (2162.5–3092.5)	3012.5 (2266.0–3490)	0.09^c^	2907.5 (2305.6–4655.6)	2416.2 (2170–3203.1)	0.05^c^	**2428.1 (2192.5–3372.5)**	**6675 (2522.5–7511.2)**	**0.039^c^**
Total number of relapses	0 (0–1)	0 (0–1)	0.94^c^	0 (0–1)	0 (0–1)	0.42^c^	**0 (0–1)**	**2 (1–2)**	**0.019^c^**

Bold values indicate statistically significant differences. ^a^Student’s unpaired *t*-test. ^b^Fisher’s exact test. ^c^Wilcoxon rank-sum test.

### Treatment response at weeks 4, 16, 28 and 40

**Fig. 2:**
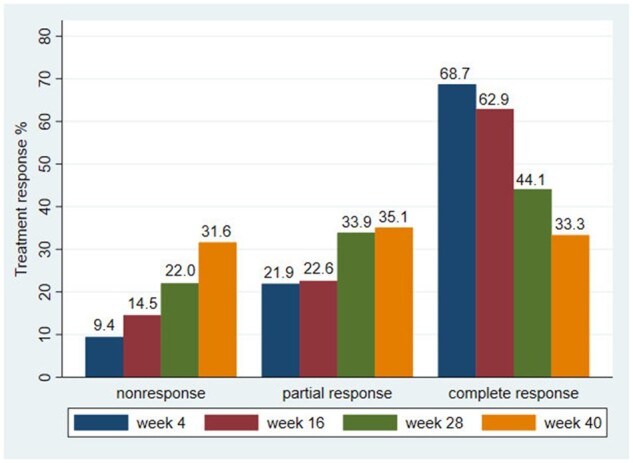
Treatment response at weeks 4, 16, 28 and 40

Results of the patients’ global VAS, ESR, CRP and duration of morning stiffness at baseline, weeks 4, 16, 28 and 40 are illustrated in Supplementary Figs S4–S7, available at *Rheumatology Advances in Practice* online. A complete response was present in 68.7, 62.9%, 44.1 and 33.3% of the patients at weeks 4, 16, 28 and 40, respectively (Fig. 2). In the subgroup analysis, the frequency of complete responses in patients with pure PMR was negligibly lower compared with the whole population (Supplementary Fig. S8, available at *Rheumatology Advances in Practice* online).

Comparison of clinical data, results of laboratory measures, TAB and ^18^F-FDG PET/CT concerning the treatment response (i.e. complete, partial and non-response) at weeks 4, 16, 28 and 40 are summarized in Table 4 and Supplementary Table S4, available at *Rheumatology Advances in Practice* online.

**Table 4:** Comparison of data with respect to the treatment response at weeks 4, 16, 28 and 40

Variables	Treatment response, week 4	Treatment response, week 16	Treatment response, week 28	Treatment response, week 40
Age	0.53^a^	**0.001^a^**	0.43^a^	0.13^a^
Gender	**0.015^b^**	0.25^b^	0.72^b^	0.78^b^
Baseline BMI	0.06^c^	0.26^c^	0.70^c^	0.51^c^
Charlson co-morbidity index	0.57^c^	0.50^c^	0.78^c^	0.37^c^
Clinical diagnosis	0.99^b^	0.63^b^	0.26^b^	0.96^b^
Pure PMR				
Pure GCA				
Concomitant PMR and GCA				
Smoking status	0.99^b^	**0.049^b^**	0.39^b^	0.72^b^
Alcohol status	0.38^b^	0.85^b^	0.50^b^	0.67^b^
Baseline ESR	0.12^c^	0.46^c^	0.24^c^	**0.013^c^**
Baseline CRP	0.99^c^	0.39^c^	0.82^c^	0.78^c^
Baseline fibrinogen	0.61^c^	0.31^c^	0.81^c^	0.90^c^
Temporal artery biopsy	0.60^b^	0.47^b^	**0.042^b^**	0.10^b^
Positive				
Negative				
^a^ ^8^F-Fluorodeoxyglucose PET/CT cut-off ≥3	0.18^b^	0.74^b^	0.38^b^	0.52^b^
Neither PMR nor GCA activity				
PMR activity				
GCA activity				
PMR and GCA activity				
^a^ ^8^F-Fluorodeoxyglucose PET/CT cut-off ≥2	0.18^b^	0.11^b^	0.61^b^	0.77^b^
Neither PMR nor GCA activity				
PMR activity				
GCA activity				
PMR and GCA activity				
PMR score	0.08^c^	0.26^c^	0.88^c^	0.93^c^
GCA score	0.62^c^	0.35^c^	0.89^c^	0.95^c^
Cumulative prednisolone dose	0.30^c^	0.44^c^	0.49^c^	0.30^c^
Total number of relapses	0.47^c^	0.17^c^	**0.026^c^**	**0.032^c^**

Bold values indicate statistically significant differences. ^a^Analysis of variance. ^b^Fisher’s exact test. ^c^Kruskal–Wallis test.

Between-groups comparison demonstrated that complete and partial responses at week 4 were significantly more frequent in female patients (complete: 46.5% in female *vs* 29.3% in male and partial: 22.4% in female *vs* 1.7% in male, *P* = 0.015) and non-response was commonly observed in male patients (20.0% in male *vs* 10% in female, *P* = 0.015).

Non-responders at week 16 were older than complete responders [76.4 (1.9) *vs* 69.5 (6.5) years, *P* = 0.001]. Although non-responders [76.4 (1.9) years] at week 16 were also older than partial responders [71.1 (9.0) years], the difference did not reach statistical significance (*P* = 0.32). There was no significant difference in age between partial and complete responders (*P* = 0.48).

TAB was positive in partial responders only at week 28 and did not differ statically between the groups (non-responders *vs* partial responders, *P* = 0.27; partial *vs* complete responders, *P* = 0.07).

At week 28, the total number of relapses during the study was higher in non-responders compared with partial [1 (1–1) *vs* 0 (0–0.5)] and complete [1 (1–1) *vs* 0 (0–1)] responders (*P* = 0.026), and no significant difference between partial and complete responders was found (*P* = 0.47). Besides, at week 40, the total number of relapses during the study was higher in non-responders compared with complete responders [1 (0–1) *vs* 0 (0–0), *P* = 0.032]. No significant differences between non- and partial responders (*P* = 0.13) or between partial and complete responders (*P* = 0.10) were observed.

Baseline ESR was lower in complete responders compared with partial responders [40 (27–60) *vs* 65.5 (46–85) mm] and non-responders [40 (27–60) *vs* 58.5 (44–83) mm] at week 40 (*P* = 0.013). There was no significant difference between partial and non-responders (*P* = 0.89).

## Discussion

The aim of the present paper was to report on the clinical features of 77 patients with newly diagnosed PMR, GCA or concomitant PMR and GCA during the 40-week study period. Whether PMR and GCA are part of the same disease entity or represent two distinct diseases is still under discussion. However, with the use of new imaging modalities [i.e. ^18^F-FDG PET/CT], signs of vasculitis in were observed ≤64% of the PMR patients, depending on the patient population (i.e. different inclusion criteria), imaging acquisitions and interpretations [13]. Patient-reported clinical symptoms, except for scalp pain and duration of morning stiffness, improved significantly following initiation of treatment, at week 4, and remained lower at week 40 compared with the relative frequencies at baseline. Contrary to the cranial symptoms, frequencies of constitutional and of shoulder girdle and hip girdle symptoms showed an increasing trend during the study period, considering that 57.3% of the patients had at least one constitutional symptom and 70.6% had one symptom in either the shoulder or hip girdle at week 40. Along with the reduction of prednisolone dose, the frequency of complete response declined from 68.7 to 33.3%, together with a rise in non-response from 9.4 to 31.6%. Several clinical features were associated with a better treatment response [i.e. female gender (week 4), younger age (week 16), fewer relapses (week 28 and 40) and lower level of baseline ESR (week 40)]. Treatment response at weeks 4, 16, 28 and 40 were independent of TAB results and fluorodeoxyglucose (FDG) uptakes on ^18^F-FDG PET/CT.

Several findings of the present study deserve further comments. In our study, most patients reported that clinical symptoms improved significantly after initiation of prednisolone treatment. Although scalp pain also improved during the study, it did not achieve statistical significance. The duration of morning stiffness did not change significantly, whereas the frequency of morning stiffness showed a significant decrease and remained lower at week 40 compared with the relative frequency at baseline. Morning stiffness is, however, reported to be weakly responsive to glucocorticoid treatment from the patients’ perspective [14]. Besides, the test–retest reliability for morning stiffness duration was found to be poor [10]. PMR activity score (PMR-AS) is the only validated index score to monitor disease activity in PMR that is based on the patient’s pain assessment, physician’s global assessment, duration of morning stiffness, the ability of the patient to elevate the arms and CRP levels [15]. Nonetheless, PMR-AS is not widely used in clinical practice and research trials for several reasons, such as inadequate description of physical function impairment by elevation of the arms and lack of consideration of other aspects of the disease, such as fatigue [16, 17]. Additionally, according to the results of the present study, the duration of morning stiffness might not be a proper indictor of the disease activity in PMR. Therefore, we believe that although PMR-AS is useful and relevant for the evaluation of patients with PMR, there is still need to develop a comprehensive tool to assess disease activity objectively and that embraces patients’ experiences.

Resolution of clinical symptoms shortly after initiation of treatment was what we expected, because glucocorticoids are potent anti-inflammatory drugs that attenuate not only the symptoms of PMR or GCA, but also other mimics of PMR/GCA, such as seronegative RA, infection, cancer and other local inflammatory diseases, which is why the diagnosis of PMR and GCA should be reassessed subsequently after initial diagnosis in several cases [18]. Nevertheless, along with the reduction of prednisolone dose, constitutional symptoms and symptoms in the shoulder or hip girdles were more commonly reported, while cranial symptoms tended to decrease. Whether presence of these symptoms, specifically constitutional symptoms, is and early sign of relapse in the patients or occurred for other reasons, such as mild glucocorticoid-induced adrenal insufficiency, cannot be distinguished.

Baseline ESR and CRP were significantly higher in patients with any cranial symptoms at week 40, although they received a significantly higher cumulative prednisolone dose during the study. These patients also had a higher number of relapses during the study period. Therefore, higher levels of acute phase reactant at baseline might suggest a subset of the patients with an intense inflammatory response who require more aggressive glucocorticoid treatment.

In the present cohort, 68.7% of the patients achieved complete response at week 4, which decreased to 62.9, 44.1 and 33.3% at weeks 16, 28 and 40, respectively. In line with our study, Matteson *et al.* [10], in a prospective cohort of 85 new-onset PMR patients who were treated with a prednisone equivalent dose of 15 mg, found that a complete response, defined in a similar manner to our study, was achieved by 53% and 56% of patients at weeks 4 and 26, respectively. In another prospective cohort study, by Hutchings *et al.* [19], on 129 PMR patients, 3 weeks after starting prednisolone 15 mg daily, 45.0% of patients achieved a complete response to therapy as defined by no pain or ≥50% improvement in pain in the shoulder and pelvic girdles, morning stiffness <30 min and normal inflammatory markers. Small differences between the results of our study and the above-mentioned studies could be explained by differences in the patient population, glucocorticoid treatment, inclusion/diagnostic criteria and definition of response to treatment. Indeed, whether a patient can truly be considered as having PMR/GCA despite lack of response to glucocorticoid is a source of concern that still needs further clarification. In our study, 9.4, 14.5, 22.0 and 31.6% of the patients had non-response at weeks 4, 16, 28 and 40, which was initially lower but subsequently higher compared with the results of the study by Matteson *et al.* [10] (16, 12 and 10% non-responders at weeks 4, 12 and 26, respectively). In light of these considerations, whether these patients represent a severe phenotype of PMR/GCA who need greater glucocorticoid doses or even DMARDs or whether an alternative diagnosis should be considered is a matter of debate. Additionally, given the dubious validity of morning stiffness duration in the present study, the definition of treatment response by using ≥70% reduction in duration of morning stiffness as one of the three above-mentioned criteria should also be called into question. In contrast, in clinical practice, it can take several weeks for acute phase reactants (i.e. ESR/CRP) to normalize fully, while PMR/GCA symptoms have responded fully to prednisolone. Given that in the present study, the patients had a thorough baseline work-up and were followed for 40 weeks, the risk of misdiagnosis is limited. We highlight, therefore, the need for developing more reliable measures to assess the treatment response in future studies.

The major strengths of our study were the prospective design and comparison of comprehensive clinical data with respect to clinical diagnosis, TAB and FDG uptakes seen on ^18^F-FDG PET/CT. Additionally, our patient population represented a real-life PMR and GCA population with varied phenotypes of the disease, which is why our study has a high degree of generalizability. However, our results should be interpreted in light of some potential limitations. Given that the numbers of patients with pure GCA or concomitant PMR and GCA were low, this can adversely affect the validity of our results. Furthermore, the duration of study follow-up (40 weeks) might be too short to determine long-term outcomes of PMR and GCA, particularly while patients are off glucocorticoid treatment.

In conclusion, the present paper reports results of several outcomes and clinical response measures in a population of newly diagnosed PMR and GCA patients during a follow-up period of 40 weeks. There is no consistency in the earlier literature with respect to optimal outcome measures, particularly patient-reported measures, for evaluating the efficacy of treatment in PMR and GCA. Despite ongoing efforts to address this issue [20], additional work is still needed to obtain valid disease-specific outcome measures that can be applied universally in the daily clinical routine and also by the future research studies.

## Supplementary Material

rkab091_Supplementary_Data
