# Supplementary material for: Clinical presentation and treatment response in patients with polymyalgia rheumatica and giant cell arteritis during a 40-week follow-up
Source: Rheumatol Adv Pract. 2021 Nov 24;5(3):rkab091. doi: 10.1093/rap/rkab091 (PMC8665449; doi:10.1093/rap/rkab091)
Supplement: rkab091_Supplementary_Data [file rkab091_supplementary_data.zip › 21-118 Supplementary Figures.pdf]

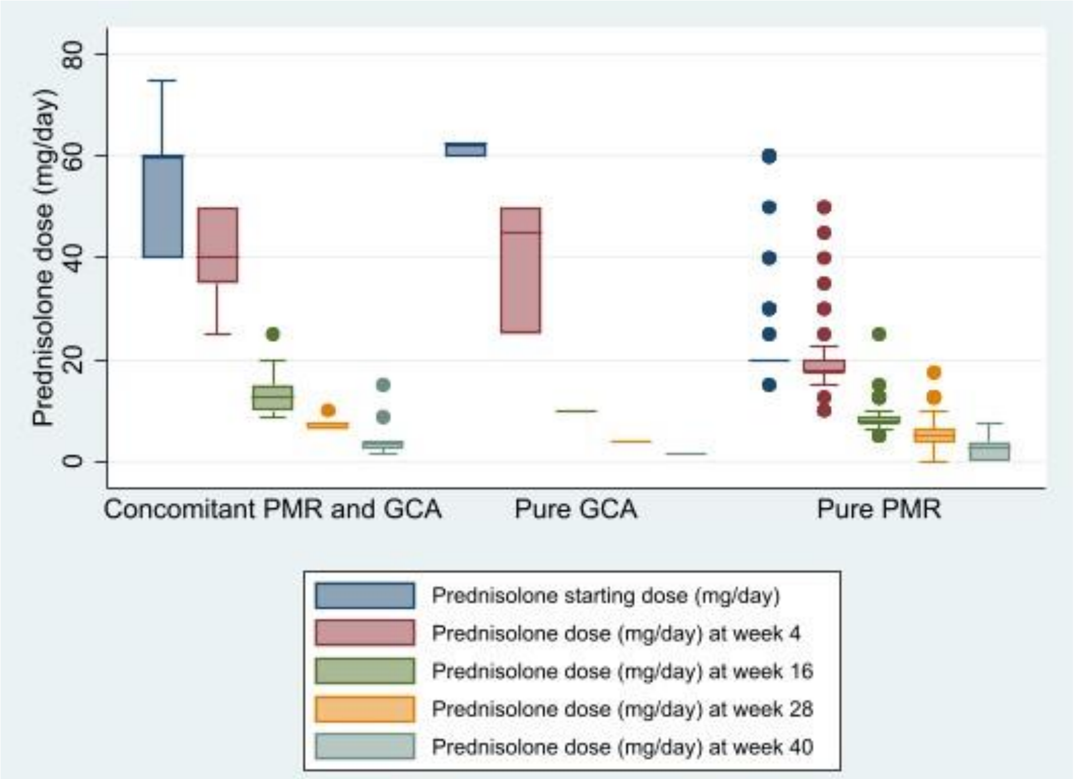


Supplementary Figure S1: The daily prednisolone dose at baseline, week 4, week 16, week 28 and week 40.

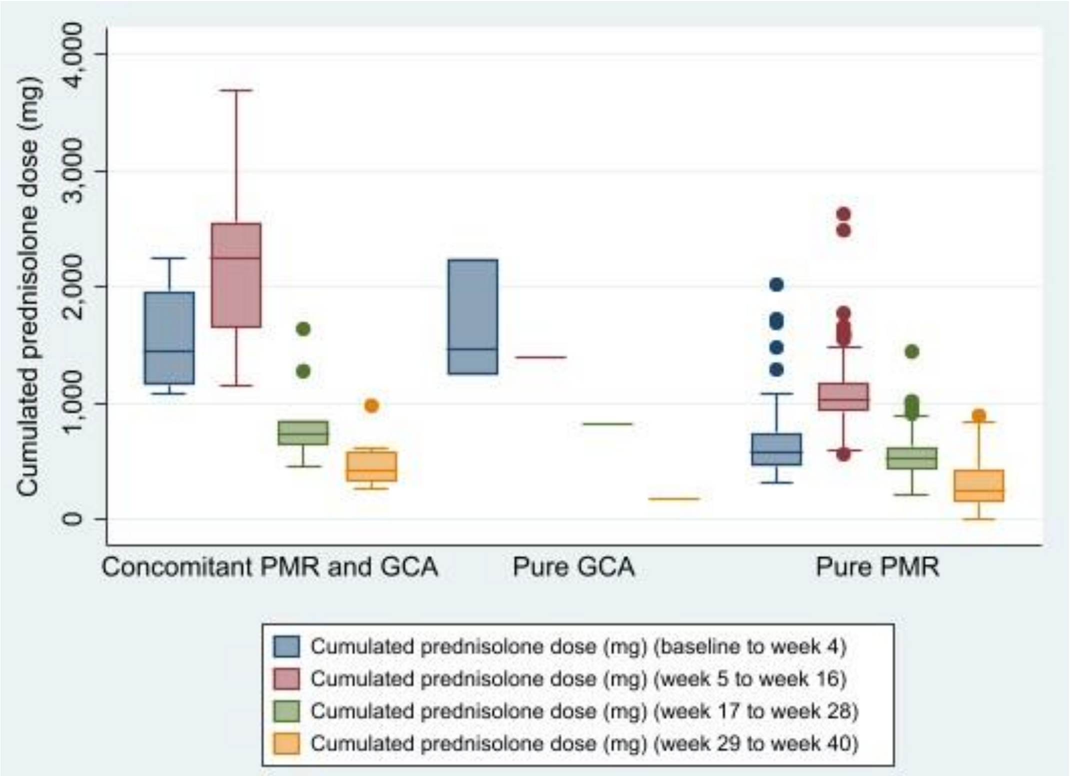


Supplementary Figure S2: The cumulated prednisolone dose (mg) during follow up.

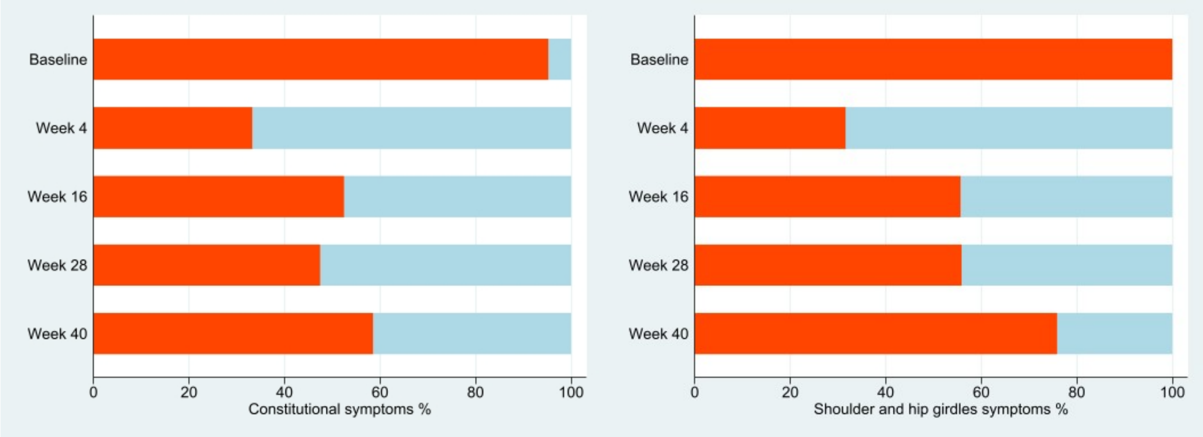


Supplementary Figure S3: Variation of clinical symptoms in patients with pure PMR during the study period.

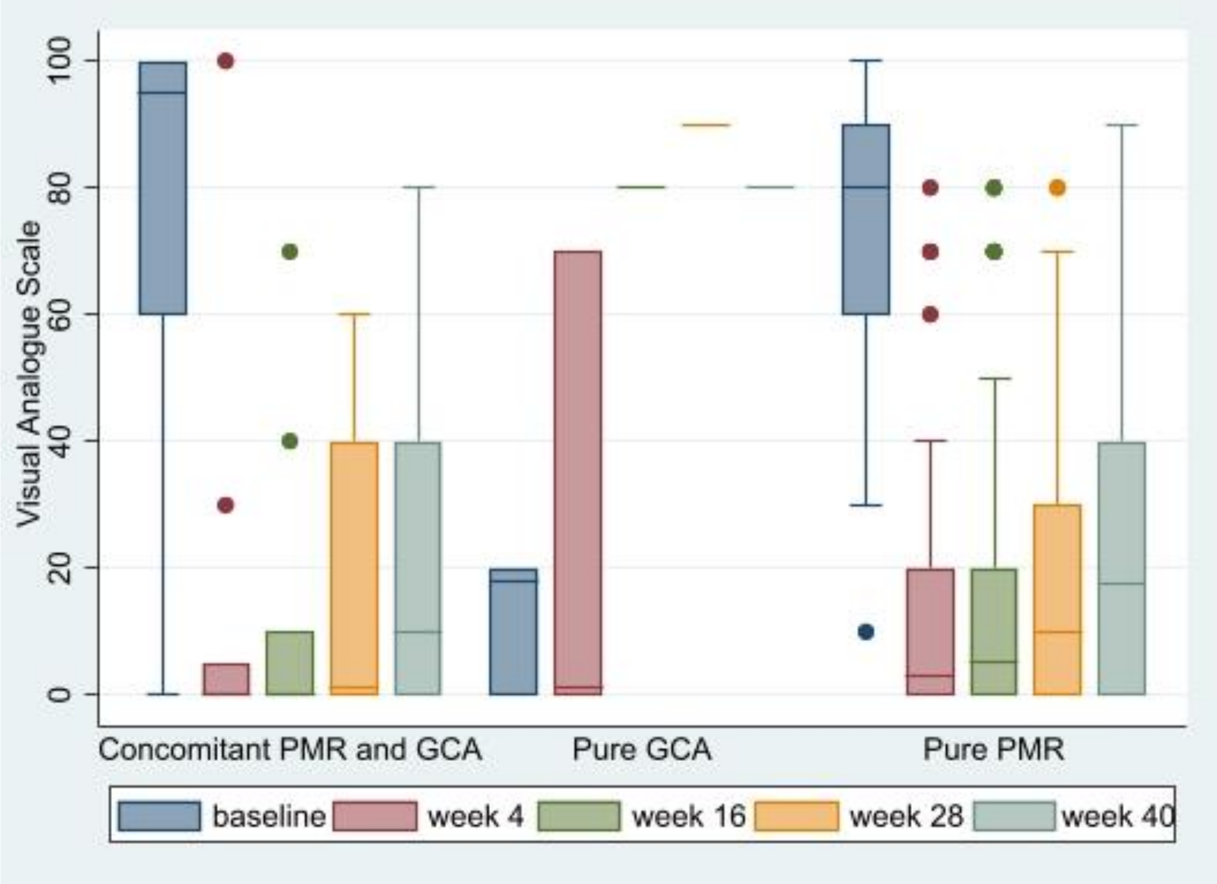


Supplementary Figure S4: Visual Analogue Scale at baseline, week 4, week 16, week 28 and week 40.

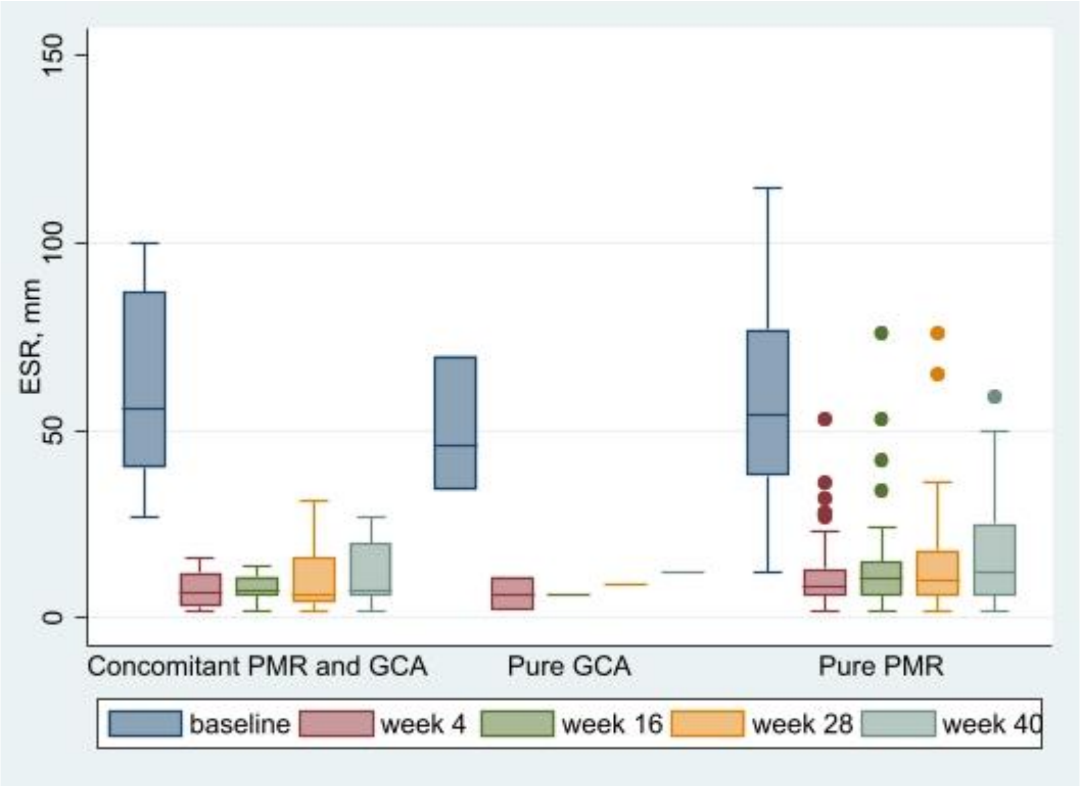


Supplementary Figure S5: ESR at baseline, week 4, week 16, week 28 and week 40.

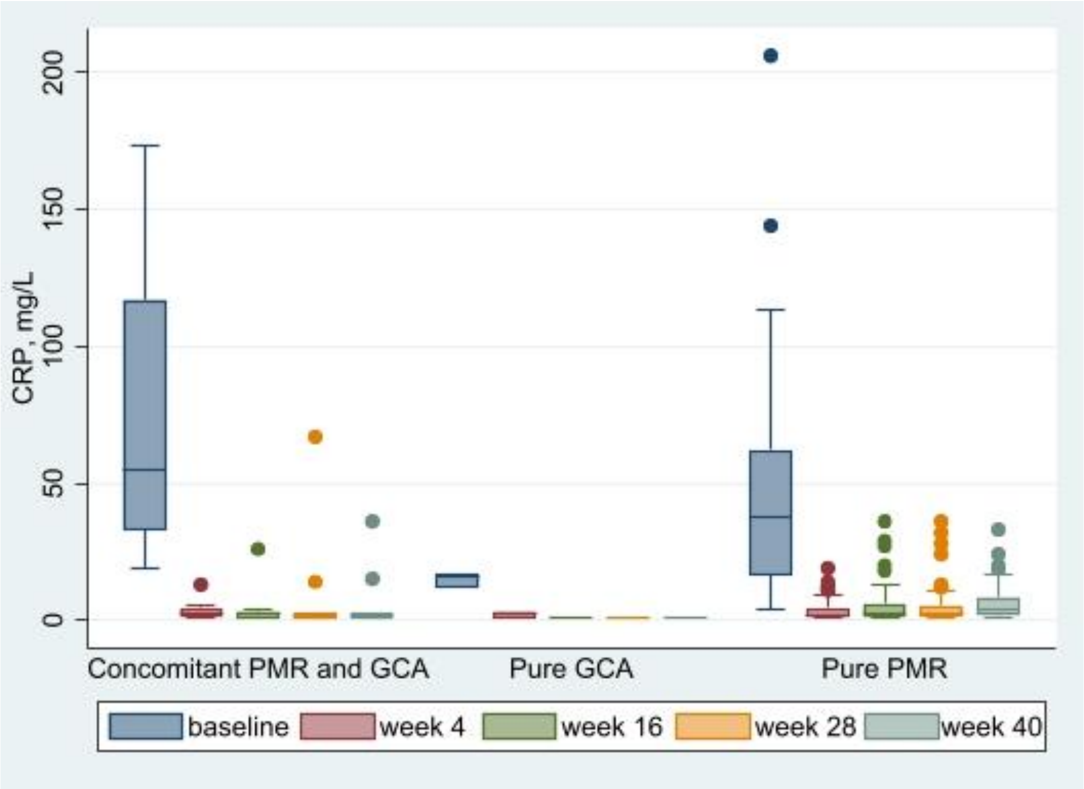


Supplementary Figure S6: CRP at baseline, week 4, week 16, week 28 and week 40.

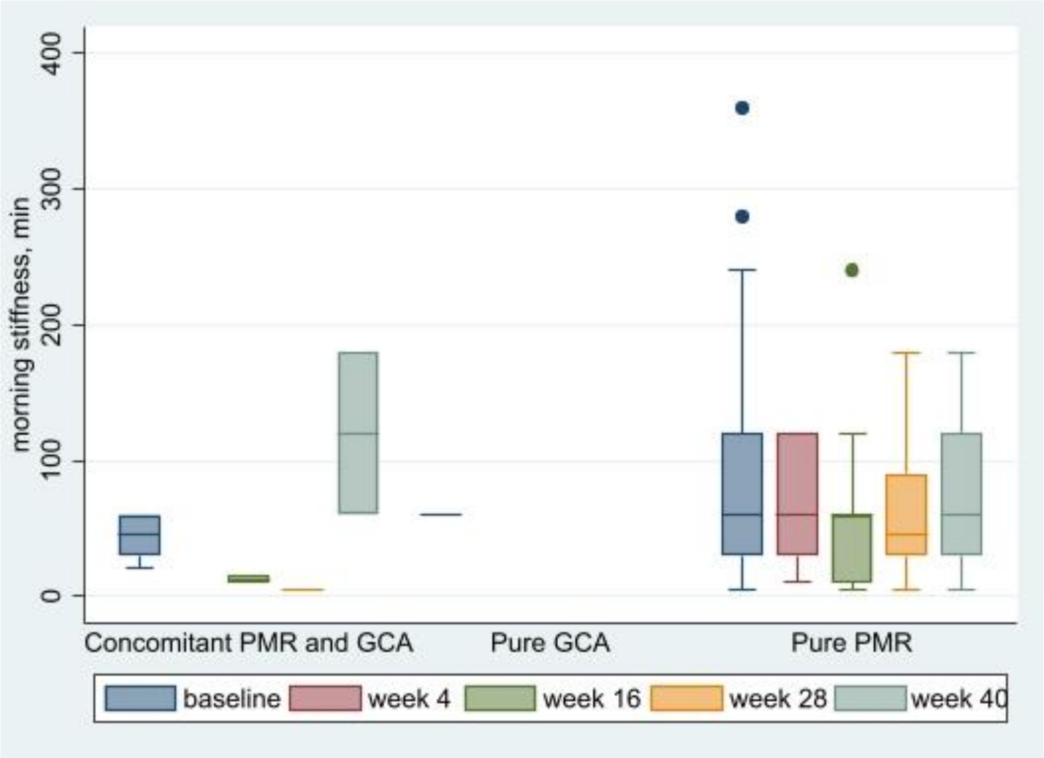


Supplementary Figure S7: Duration of morning stiffness at baseline, week 4, week 16, week 28 and week 40.

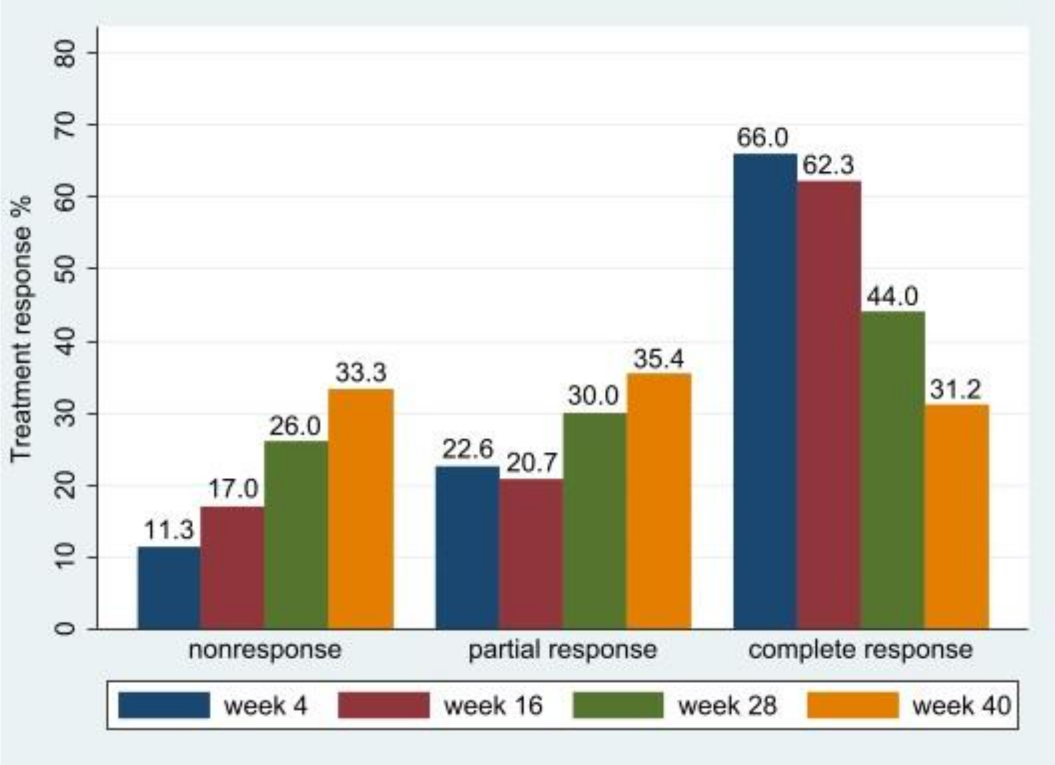


Supplementary Figure S8: Treatment response in patients with pure PMR at week 4, week 16, week 28 and week 40.
