# Supplementary material for: Clinical presentation and treatment response in patients with polymyalgia rheumatica and giant cell arteritis during a 40-week follow-up
Source: Rheumatol Adv Pract. 2021 Nov 24;5(3):rkab091. doi: 10.1093/rap/rkab091 (PMC8665449; doi:10.1093/rap/rkab091)
Supplement: rkab091_Supplementary_Data [file rkab091_supplementary_data.zip › 21-118_Supplementary_Tables.docx]

| **Supplementary Table S1: Summary of patients reported symptoms in PMR (n=64) at baseline, week 4, week 16, week 28 and week 40.** | | | | | |
| --- | --- | --- | --- | --- | --- |
| **Symptoms** | **Baseline (n=64)** | **Week 4 (n=61)** | **Week 16 (n=61)** | **Week 28 (n=60)** | **Week 40 (n=58)** |
| **Constitutional, n (%)** | | | | | |
| Weight loss | 21 (32.8) | **5 (8.2)**  **0.001^1^** | **2 (3.3)**  **<0.001^1^** | **1 (1.7)**  **<0.001^1^** | **6 (10.3)**  **0.005^1^** |
| Tiredness | 60 (93.7) | **19 (31.7)**  **<0.001^1^** | **30 (49.2)**  **<0.001^1^** | **27 (45.0)**  **<0.001^1^** | **31 (53.4)**  **<0.001^1^** |
| Fever | 10 (15.6) | **0 (0)**  **0.002^1^** | **3 (4.9)**  **0.002^1^** | **1 (1.7)**  **0.003^1^** | **0 (0)**  **0.002^1^** |
| **Shoulder girdle, n (%)** | | | | | |
| Pain in neck | 41 (65.1) | **5 (8.2)**  **<0.001^1^** | **12 (19.7)**  **<0.001^1^** | **7 (11.7)**  **<0.001^1^** | **12 (20.7)**  **<0.001^1^** |
| Pain in shoulder | 59 (92.2) | **6 (9.8)**  **<0.001^1^** | **13 (21.3)**  **<0.001^1^** | **10 (16.7)**  **<0.001^1^** | **22 (37.9)**  **<0.001^1^** |
| Pain in upper arm | 59 (92.2) | **6 (10)**  **<0.001^1^** | **13 (21.3)**  **<0.001^1^** | **14 (23.3)**  **<0.001^1^** | **16 (37.6)**  **<0.001^1^** |
| **Hip girdle, n (%)** | | | | | |
| Pain in buttock | 55 (85.9) | **8 (13.1)**  **<0.001^1^** | **14 (22.9)**  **<0.001^1^** | **13 (21.7)**  **<0.001^1^** | **17 (29.8)**  **<0.001^1^** |
| Pain in thigh | 58 (92.1) | **6 (9.8)**  **<0.001^1^** | **14 (22.9)**  **<0.001^1^** | **14 (23.3)**  **<0.001^1^** | **16 (27.6)**  **<0.001^1^** |
| **Morning stiffness, n (%)** | 56 (87.50 | **6 (9.8)**  **<0.001^1^** | **14 (22.9)**  **<0.001^1^** | **25 (42.4)**  **<0.001^1^** | **30 (51.7)**  **<0.001^1^** |
| **Morning stiffness, min** | 60 (30-120) | 60 (30-120)  0.32^2^ | 60 (10-60)  0.33^2^ | 45 (30-90)  0.36^2^ | 60 (30-120)  0.78^2^ |
| 1. McNemar’s test  2. Wilcoxon signed rank test  *P-values relate to hypothesis testing on the difference between baseline and follow-up data.  § Bold values indicate statistical significance differences. | | | | | |

| **Supplementary Table S2: Summary of physical examination in patients with PMR (n=64) at baseline, week 4, week 16, week 28 and week 40.** | | | | | |
| --- | --- | --- | --- | --- | --- |
| **Physical Examination, n %** | **Baseline** | **Week 4** | **Week 16** | **Week 28** | **Week 40** |
| Neck tenderness to palpation | 12 (21.8) | **2 (3.3)**  **0.004^*^** | **2 (3.4)**  **0.002** | **2 (3.3)**  **0.002** | **2 (3.5)**  **0.007** |
| Shoulder tenderness to palpation | 18 (32.7) | **3 (4.9)**  **<0.001** | **4 (6.8)**  **0.001** | **3 (5.0)**  **<0.001** | **2 (3.5)**  **<0.001** |
| Upper arm tenderness to palpation | 25 (45.4) | **2 (3.3)**  **<0.001** | **3 (5.1)**  **<0.001** | **6 (10.0)**  **<0.001** | **3 (5.3)**  **<0.001** |
| Active shoulder abduction (0-180)°  Not at all  Yes, hardly  Yes, effortless | 18 (32.1)  35 (62.5)  3 (5.4) | **0 (0)**  **4 (6.9)**  **54 (93.1)**  **<0.001** | **1 (1.7)**  **1 (1.7)**  **58 (96.7)**  **<0.001** | **2 (3.3)**  **3 (5.0)**  **55 (91.7)**  **<0.001** | **2 (3.5)**  **7 (12.3)**  **48 (84.2)**  **<0.001** |
| Buttock tenderness to palpation | 20 (36.4) | **4 (6.6)**  **<0.001** | **5 (8.5)**  **<0.001** | **1 (1.7)**  **<0.001** | **0 (0)**  **<0.001** |
| Thigh tenderness to palpation | 21 (38.2) | **4 (6.6)**  **<0.001** | **5 (8.5)**  **0.001** | **1 (1.7)**  **<0.001** | **2 (3.5)**  **<0.001** |
| *P-values are calculated by McNemar’s test and relate to hypothesis testing on the difference between baseline and follow-up data.  § Bold values indicate statistical significance differences. | | | | | |

| **Supplementary Table S3: Comparison of data in PMR patients with and without constitutional, shoulder and hip girdles symptoms at week 40.** | | | | | | |
| --- | --- | --- | --- | --- | --- | --- |
|  | Week 40 | | | | | |
| **Variables** | **Any constitutional symptoms** | | | **Any shoulder and hip girdles symptoms** | | |
|  | No | Yes | p-value | No | Yes | p-value |
| Age, y±SD | 72.3±6.8 | 71.8±7.3 | 0.77^1^ | 72.4±7.6 | 71.9±7.0 | 0.80^1^ |
| Female gender, n (%) | 13 (22.4) | 22 (37.9) | 0.59^2^ | 8 (13.8) | 27 (46.5) | 0.99^2^ |
| Baseline BMI, kg/m^2^ | 23.9 (21.7-26.9) | 26.1 (23-28.3) | 0.14^3^ | 24.6 (21.4-27.9) | 25.5 (22.6-28.1) | 0.37^3^ |
| Charlson comorbidity index | 3 (2-4) | 3 (3-4) | 0.34^3^ | **4 (3-5)** | **3 (2-4)** | **0.014^3§^** |
| Smoker (including former smoker), n (%) | 14 (24.1) | 25 (43.1) | 0.26^2^ | 9 (15.5) | 30 (51.7) | 0.99^2^ |
| Alcohol > 6 units per week | 6 (10.3) | 10 (17.2) | 0.77^2^ | 3 (5.2) | 13 (22.4) | 0.74^2^ |
| Baseline ESR, mm [normal range: 2-20] | 55.2±22.6 | 58.1±23.8 | 0.65^1^ | 55.5±25.7 | 57.2±22.6 | 0.82^1^ |
| Baseline CRP, mg/L [normal range: <6.0] | 41.5 (23.5-64.5) | 33 (14-60) | 0.29^3^ | 56 (31-70) | 31 (16-59) | 0.08^3^ |
| Baseline Fibrinogen, µmol/L [normal range: 5.2-12.6] | 15.2 (13.0-17.7) | 14 (12.8-17.5) | 0.60^3^ | 15.6 (13.3-18.0) | 14 (12.9-17.4) | 0.45^3^ |
| Temporal Artery Biopsy, n (%)  Positive  Negative | 0 (0)  21 (40.4) | 2 (3.8)  29 (55.8) | 0.51^2^ | 0 (0)  12 (23.1) | 2 (3.8)  38 (73.1) | 0.99^2^ |
| 18F-FDG PET/CT cut off ≥3, n (%)  Neither PMR nor GCA activity  PMR activity | **0 (0)**  **24 (42.1)** | **11 (19.3)**  **22 (38.6)** | **0.001^2^** | 2 (3.5)  12 (21.05) | 9 (15.8)  34 (59.6) | 0.71^2^ |
| 18F-FDG PET/CT cut off ≥2, n (%)  Neither PMR nor GCA activity  PMR activity | 0 (0)  22 (41.5) | 4 (7.5)  27 (50.9) | 0.13^2^ | 1 (1.9)  13 (24.5) | 3 (5.7)  36 (67.9) | 0.99^2^ |
| PMR score | 16 (13.5-17) | 14 (10-17) | 0.13^3^ | 16 (12-17) | 14 (11-17) | 0.39^3^ |
| Cumulated prednisolone dose, mg | **2210 (2149.4-2406.9)** | **2497.5 (2205-3230)** | **0.01^3^** | 2425.6 (2266.0-3475) | 2355 (2166.2-3140.6) | 0.33^3^ |
| Total numbers of relapse | 0.5 (0-1) | 0 (0-1) | 0.68^3^ | 0 (0-1) | 1 (0-1) | 0.36^3^ |
| 1- Student's t-test  2. Fisher's exact test  3. Wilcoxon rank-sum test  § Bold values indicate statistical significance differences.  BMI: Body Mass Index, ESR: Erythrocyte Sedimentation Rate, CRP: C-reactive protein | | | | | | |

| **Supplementary Table S4: Comparison of data in PMR patients with respect to the treatment response at weeks 4, 16, 28 and 40.** | | | | |
| --- | --- | --- | --- | --- |
| **Variables** | **Treatment response week 4** | **Treatment response week 16** | **Treatment response week 28** | **Treatment response week 40** |
| Age | 0.87^1^ | **0.012^1§^** | 0.95^1^ | 0.66^1^ |
| Gender | **0.029^2^** | 0.28^2^ | 0.57^2^ | 0.66^2^ |
| Baseline BMI | 0.09^3^ | 0.45^3^ | 0.52^3^ | 0.30^3^ |
| Charlson comorbidity index | 0.26^3^ | 0.32^3^ | 0.75^3^ | 0.40^3^ |
| Smoking status | 0.91^2^ | 0.14^2^ | 0.16^2^ | 0.86^2^ |
| Alcohol status | 0.57^2^ | 0.67^2^ | 0.60^2^ | 0.99^2^ |
| Baseline ESR | 0.79^1^ | 0.92^1^ | 0.68^1^ | 0.55^1^ |
| Baseline CRP | 0.93^3^ | 0.93^3^ | 0.66^3^ | 0.99^3^ |
| Baseline Fibrinogen | 0.98^3^ | 0.28^3^ | 0.58^3^ | 0.94^3^ |
| Temporal Artery Biopsy  Positive  Negative | 0.99^2^ | 0.17^2^ | 0.54^2^ | 0.64^2^ |
| 18F-FDG PET/CT cut off ≥3  Neither PMR nor GCA activity  PMR activity | 0.05^2^ | 0.21^2^ | 0.10^2^ | 0.19^2^ |
| 18F-FDG PET/CT cut off ≥2  Neither PMR nor GCA activity  PMR activity | 0.17^2^ | **0.016^2^** | 0.15^2^ | 0.38^2^ |
| PMR score | 0.06^3^ | 0.12^3^ | 0.71^3^ | 0.71^3^ |
| Cumulated prednisolone dose | 0.07^3^ | 0.08^3^ | 0.22^3^ | 0.14^3^ |
| Total numbers of relapse | 0.68^3^ | 0.21^3^ | **0.021^3^** | **0.025^3^** |
| 1. Analysis of variance  2. Fisher's exact test  3. Kruskal-Wallis  § Bold values indicate statistical significance differences.  BMI: Body Mass Index, ESR: Erythrocyte Sedimentation Rate, CRP: C-reactive protein | | | | |
